# An intervention to determine the effectiveness of the *Sanubari* optimal health program (OHP) in improving mental well-being among junior doctors in Malaysia: a quasi-experimental study

**DOI:** 10.1186/s12889-024-20100-z

**Published:** 2024-09-27

**Authors:** Mohamad Aznuddin Abd Razak, Umi Adzlin Silim, Aida Farhana Suhaimi, Siti Salwa Ramly, Nurul Nadia Ismail, Adlin Mohd Salleh, Aina Waheeda Mohamad

**Affiliations:** 1 Institute for Public Health, National Institutes of Health, Ministry of Health Malaysia, Setia Alam, 40170 Selangor Malaysia; 2 Department of Psychiatry and Mental Health, Hospital Sultan Idris Shah, Serdang, 43400 Selangor Malaysia; 3 Psychology Department, Medcare and Camali Clinic Dubai, P.O. Box 505294, Dubai, UAE; 4 Department of Psychiatric and Mental Health, Hospital Sultan Abdul Halim, Sungai Petani, 08000 Kedah Malaysia; 5 Department of Psychiatric and Mental Health, Hospital Sultanah Bahiyah, Alor Setar, 05460 Kedah Malaysia; 6 Department of Psychiatric and Mental Health, Hospital Tengku Ampuan Rahimah, Klang, 41200 Selangor Malaysia; 7 Clinical Research Centre, Hospital Kuala Lumpur, Jalan Pahang, 50586 Kuala Lumpur Malaysia

**Keywords:** *Sanubari*, Optimal Health Program (OHP), Junior doctor, Mental health, Well-being

## Abstract

**Background:**

Mental health problems among junior doctors in Malaysia pose a significant concern, as they not only adversely affect their overall well-being but also impact the quality of health services they provide. Therefore, it is important to implement interventions aimed at preserving their mental health. This study aimed to evaluate the effectiveness of the *Sanubari* Optimal Health Program (OHP) intervention in enhancing the mental well-being of junior doctors in Malaysia.

**Methods:**

The study utilised a quasi-experimental design involving 204 junior doctors who participated in the intervention. The *Sanubari* OHP Intervention Package was employed alongside self-administered questionnaires for mental health and well-being assessment. To evaluate the effects over time, repeated measures ANOVA was employed to analyse within-group and between-group changes in various endpoints, as measured at different assessment time points.

**Results:**

The study revealed that self-efficacy and adaptive coping behaviours scores increased over time among the intervention group and decreased among the control group. Yet, depression and anxiety scores decreased significantly over time among the intervention group but not in the control group. No significant differences were observed in well-being and maladaptive coping strategies among the groups.

**Conclusions:**

This study showed promising results regarding the effectiveness of *Sanubari* OHP in improving mental well-being among junior doctors.

## Background

Mental health is a state of mental well-being that enables people to cope with the stress of life, realize their abilities, learn well and work well, and contribute to their community [1]. A combination of individual, social, and structural factors can influence mental health, either safeguarding it or posing risks, ultimately influencing where a person stands on the mental health spectrum [1]. As a crucial component of holistic well-being, mental health profoundly impacts individuals across all professions, including those within the medical field especially junior doctors.

In Malaysia, junior doctors are known as ‘House Officers’. Housemanship training, also referred to as an internship, is a mandatory period of supervised practical training for medical graduates, as stipulated by the Medical Act 1971. This training, which lasts a minimum of one year, involves rotating through various departments to gain hands-on experience in patient care under the supervision of senior doctors. The objective is to ensure that medical graduates develop the requisite skills and knowledge to practice competently. During this period, junior doctors must complete six postings in five core departments which are medicine, surgery, obstetrics and gynaecology, paediatrics, and orthopaedics. Additionally, they are required to undertake one posting in a speciality area such as anaesthesiology, emergency medicine, psychiatry, or primary care [2].

Throughout their training, medical students and junior doctors encounter heightened personal and professional stressors. They often face unique challenges and stressors that can significantly impact their mental health [3]. The demanding nature of their work, long hours and time constraints, exposure to traumatic experiences, and the burden of responsibility can take a toll on their mental health [4]. These mental health challenges can have severe consequences, leading to decreased job satisfaction, impaired professional performance, medical errors, and decreased quality of patient care [4]. The stigma surrounding mental health within the medical community often prevents these doctors from seeking help, exacerbating the problem further [5].

These mental health issues among doctors require urgent attention, support and intervention. Mental health interventions must begin before the onset of mental disorders. Addressing mental health among doctors by providing doctors with the necessary support systems, resources, and strategies to prioritize their mental well-being is essential for promoting a healthy and sustainable medical workforce. This includes implementing comprehensive mental health programs, destigmatizing mental health discussions, improving work-life balance, and fostering a culture of self-care and resilience [6]. Studies have shown the effectiveness of various mental health interventions. For instance, a self-help intervention improved self-efficacy, perceived stress, mental distress, and self-reported levels of mindfulness among medical students at a Malaysian university within the first week [7]. Phang et al. reported that five weeks of Mindfulness-based Intervention significantly reduced perceived stress and mental distress at 1-week post-intervention with a medium effect size [7]. Another study evaluated the effectiveness of a mailed intervention to reduce distress among ‘at-risk’ general practitioners and also found a reduction of the average GHQ-12 score from 6.06 in the control group to 4.06 with a difference of 2.00 and from 6.20 to 2.76 for the intervention group, a difference of 3.44. There was a substantial reduction in psychological distress (*P* = 0.03) when the means were compared [8].

The Optimal Health Program (OHP), developed by St Vincent’s Hospital in Melbourne, Australia, in 2001, is another self-management intervention designed to promote self-efficacy and optimal well-being [9]. OHP encourages individuals to engage in reflective conversations, write down ideas, ask questions, and develop plans to support their well-being. This strength-based program helps participants define their health, recognize strengths and vulnerabilities, identify stressors, build strategies, learn about healthy lifestyles, enhance support systems, set goals, and prepare health plans. The OHP was developed based on five architectures: situational awareness optimal health, enhancing self-efficacy “I can do” model, determinants of health factors of well-being, enhancing change visioning and goal setting, and summarising learning and sustain plans [10]. The original modules are delivered in eight weekly sessions, either individually or in groups, with optional booster sessions.

Initially, research on OHP was predominantly within mental healthcare settings, but it has since extended to the general community with a greater focus on well-being rather than illness [9, 11–15]. The OHP has proven effective in improving health and social functioning in patients with mental health problems [9]. The OHP has provided a promising framework for enhancing Malaysia’s mental healthcare services and a valuable platform for integrating psychosocial care within primary healthcare [14]. OHP’s initial engagement and training among public health sectors and NGOs have laid the groundwork for future implementation and delivery in Malaysia [10]. A forward-backwards translation and cultural adaptation of OHP was conducted in Malaysia. This adapted Malay version has shown effectiveness in improving overall health well-being among COVID-19 patients, nurses during the pandemic, and diabetic patients [16–18]. Thus, the findings from these studies may suggest similar effectiveness among junior doctors. Therefore, this study aims to determine the effectiveness of OHP in improving the mental well-being of junior doctors in Malaysia.

## Methods

### Design and sampling

This study was a quasi-experimental design that targeted junior doctors who have been working for more than 6 weeks during recruitment in public hospitals. Universal sampling was applied to select the sample, meaning all eligible junior doctors who met the inclusion criteria were included in the study. The inclusion criteria were as follows: [1] junior doctors who had been working for more than 6 weeks, and [2] junior doctors who provided informed consent to participate in the study. We selected junior doctors with more than 6 weeks of working experience to ensure that participants have had sufficient exposure to the demands and stressors of the job. Typically, junior doctors undergo an initial 2-week “tagging” period, where they are orientated and gradually introduced to their responsibilities. Following this, a minimum of one month of active work allows them to experience the full scope of their duties, including the pressures and challenges inherent to the role. By setting the inclusion criterion more than 6 weeks, we aim to capture participants who have moved beyond the initial adjustment phase and are more likely to have encountered the typical stressors associated with the job, providing a more accurate assessment of their mental health and well-being. While, the exclusion criteria were junior doctors who were diagnosed with mental illness, determined by asking if they were on medication for mental illness before being selected for the study.

### Sample size and selection

We used G-Power sample size calculator to estimate sample size for 2 independent means [19] based on the mean of wellbeing from previous study; intervention group (M = 70.4, SD = 19.62) and control group (M = 76.65, SD = 15.74) [18]. The estimated sample size is 101 subjects in each arm after taking into consideration of effect size = 0.35, power of 0.8, and alpha level of 0.05. After adjusting to 10% non-response or due to loss of follow-up, the total sample size needed was 112. Therefore, the overall sample size required for both arms was 224 subjects (112 intervention, 112 control). We round off the number to 230 subjects.

Ten hospitals were chosen based on the availability of the OHP facilitator. Five hospitals were listed as intervention hospitals, and another five were listed as control hospitals. We matched the control hospital with the intervention hospital based on comparable size and geographic location. As a sampling frame, a list of medical junior doctors with a service duration of 6 to 12 weeks from each intervention and control hospital was obtained. A total of 23 medical junior doctors from each hospital were selected from the list.

### Intervention

In a preliminary study that assessed the need for OHP in Malaysia, the OHP provided a promising framework for building the capacity of local mental healthcare services. Following a process of translation and cultural adaptation, the Malaysian OHP Program was developed by technical and language experts who translated forward and backward [10]. The adapted Malay OHP was named *Pohon Sihat* and then changed to *Sanubari* OHP. The *Sanubari* OHP was delivered in six sessions: five weekly sessions followed by one booster session (optional) after three months. The sessions followed this sequence: “Optimal Health,” “I-Can-Do Model,” “Health Factors of Wellbeing,” “Visioning and Goal Setting,” and “Health Plan.”

### Implementation of data collection

**Fig. 1:**
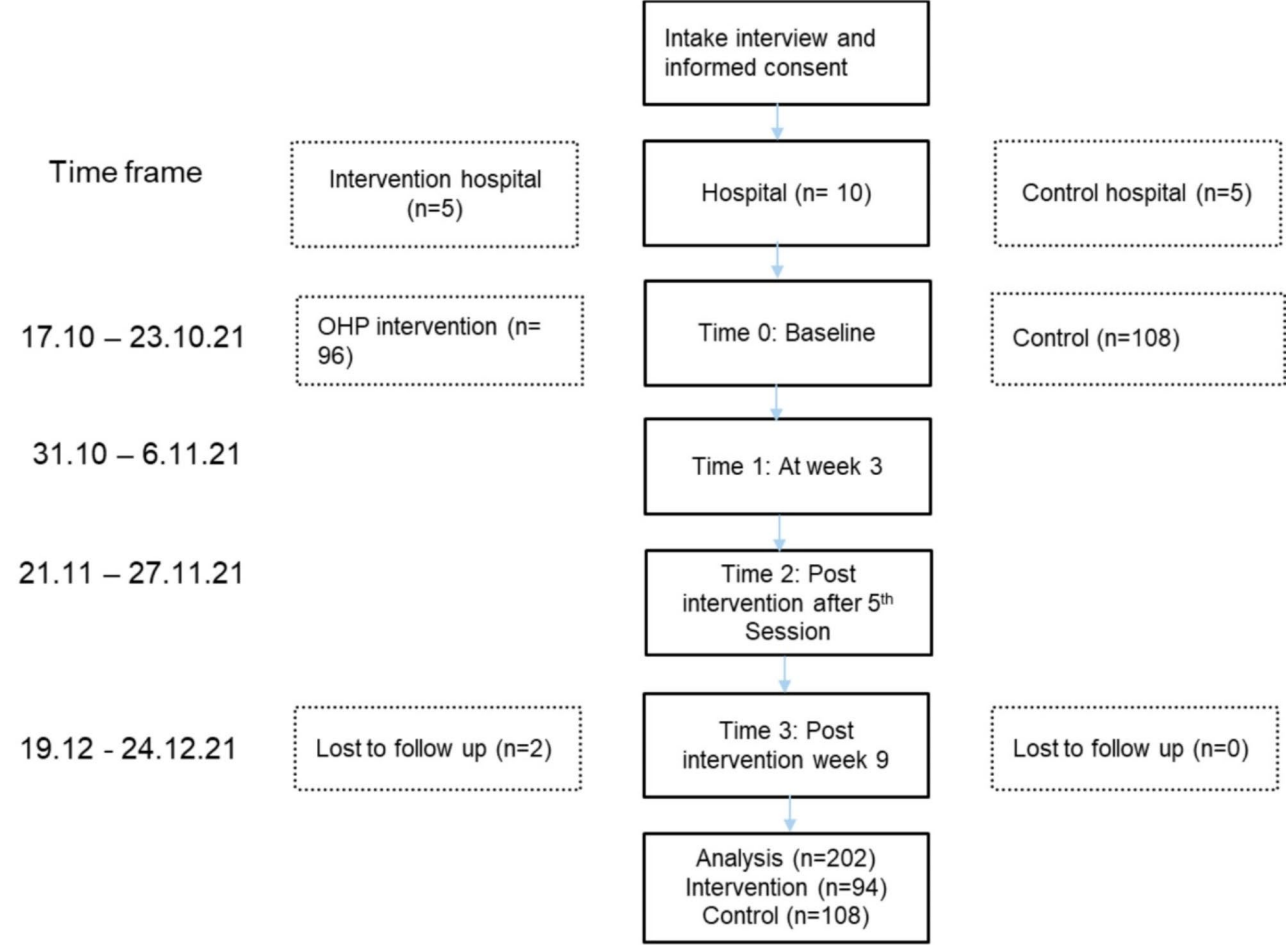
Consort flow diagram of implementation for *Sanubari* Intervention among junior doctors

This study involved ten selected hospitals (five intervention and five control hospitals). Junior doctors from intervention hospitals received five sessions of *Sanubari* OHP intervention (one session every week), while junior doctors from the control hospitals did not receive any treatment within the period. Both the control and intervention groups were required to complete a series of questionnaires before intervention as the baseline assessment. After the third session, intervention and control groups again completed the series of questionnaires mailed to them. The post-intervention was performed twice using the same questionnaires, one right after the fifth *Sanubari* OHP session and one after week nine from the baseline (Fig. 1). Concurrently, the re-assessment was performed on the control group. The intervention was done virtually using an online platform (Microsoft Team) and by trained facilitators consisting of psychiatrists, clinical psychologists, and medical officers from the psychiatry and mental health departments. During the video conferencing sessions conducted via Microsoft Teams, all participants and facilitators are required to be on camera throughout the session. To ensure that participants remain engaged and attentive, the facilitators frequently check in with them for responses. The sessions are designed to be highly interactive, incorporating engaging content such as videos and animations, as well as interactive tools like polls and quizzes. These tools help maintain participant engagement and ensure active participation throughout the session. Each session lasts approximately 90 min. The sessions are conducted in groups, with each group consisting of around 10 to 12 participants. Each session is facilitated by two trained *Sanubari* OHP facilitators. Throughout the program, each group attends a total of 5 sessions.

### Assessment tools

A set of questionnaires included a sociodemographic profile (gender, age, ethnicity, hospital), and the validated Patient Health Questionnaire (PHQ-9), General Anxiety Disorders (GAD-7), WHO-5 Well-being Index (WHO-5), General Self-Efficacy Scale (GSE) and Brief COPE was used for participant’s assessment.

The PHQ-9 is a nine-item self-administered measurement to assess depression symptoms and the impairments related to the symptoms. Participants rate items on a 4-point Likert scale ranging from 0 to 3. The sum of all items ranges between 0 and 27. The higher the score, the worse of the symptoms. The Malay validated PHQ-9 has a Cronbach’s α estimate of 0.70, sensitivity of 87% and specificity of 82% [20].

GAD-7 is a seven-item self-administered measurement that assesses the presentation of anxiety symptoms and the impairments related to the symptoms. Participants rate items on a 4-point Likert scale ranging from 0 to 3. The sum of all items ranges between 0 and 21. Higher score showed the symptoms of anxiety is worse. The Malay validated GAD-7 has a Cronbach’s α estimate of 0.74, sensitivity of 76% and specificity of 94% [21].

WHO-5 is a five-item self-administered measurement that assesses emotional well-being and mental health [22]. Participant’s rate items on a 5-point Likertscale ranging between 0 (none of the time) and 5 (all of the time). The raw score that ranges from a minimum of 0 (absence of well-being) to a maximum of 25 (maximum well-being) are then multiplied by four to obtain the percentage scale.

The General Self-Efficacy (GSE) is a 10-item uni-dimensional scale for assessing a general sense of perceived self-efficacy to predict coping with daily hassles and adaptation after experiencing all kinds of stressful life events [23]. Responses are given on a four-point scale with scores ranging from 10 to 40. Examples of items include ‘‘I can always solve difficult problems if I try hard enough.’’ Participants respond to each item using a 4-point scale ranging from 1 (not at all true) to 4 (exactly true). Higher scores indicate higher levels of self-efficacy. The Cronbach’s alpha value in this study was 0.89.

Brief COPE is a 28-item that rated under 4 categories of responses in identifying coping strategies. The responses were: 1 - I haven’t been doing this at all, 2 - I have been doing this for a little bit, 3 - I have been doing this a medium amount, and 4 - I have been doing this a lot. Each specific item score was treated as continuous data whereby the mean score for every 14 different constructs was measured. The 14 coping strategies that can be identified from 28 items in the questionnaire which are; self-distraction (items 1 and 19), active coping (items 2 & 7), denial (items 3 & 8), substance use (items 4 & 11), use of emotional support (items 5 & 15), use of instrumental support (items 10 & 23), behavioural disengagement (items 6 & 16), venting (items 9 & 21), positive reframing (items 12 & 17), planning (items 14 & 25), humour (items 18 & 28), acceptance (items 20 & 24), religion (items 22 & 27), and self-blame (items 13 & 26). We categorised 14 coping strategies into two types of coping behaviour: adaptive coping strategies (active coping, use of instrumental support, positive reframing, planning, acceptance, and religion) and maladaptive coping strategies (denial, substance use, venting, humour, self-blame). The original version of the questionnaire was validated in Malay in 2011, and Cronbach’s alpha was 0.83 [24].

### Statistical analyses

The data was managed and analysed using the SPSS for Windows version 26.0 [25]. Categorical variables were summarised as frequencies and percentages. On the other hand, numerical variables were summarized as means and standard deviations. The repeated measures ANOVA was used to assess the within- and between-group effects in all the endpoints according to the time points of assessments set. All the analyses were one-tailed, and the significant value was 5%. Intention-to-treat analysis was performed to resolve the issue of missing data due to loss of follow-up.

## Results

### Baseline (T0) characteristics of participants

A total of 230 junior doctors were recruited, and 204 of them agreed to participate in the study from both intervention and control hospital groups at baseline (T0). Table 1 shows that 47.1% of participants were in the intervention hospital group and 52.9% were in the control hospital group. Both groups showed that the majority of the participants at baseline were female.

**Table 1:** Sample characteristics at baseline (T0) for both intervention and control groups

Characteristics	Intervention		Control	
	*n*	Percentage	*n*	Percentage
Overall	96	47.1	108	52.9
Sex				
Male	34	35.4	37	34.3
Female	62	64.6	71	65.7
Department				
Medical	25	26.0	18	16.7
Obstetrics & Gynaecology	14	14.6	30	27.8
Orthopaedics	19	19.8	18	16.7
Paediatrics	17	17.7	24	22.2
Surgical	21	21.9	18	16.7

### The mean difference at baseline, T0

Overall, 204 junior doctors answered the online assessments (96 from the intervention group and 108 from the control group). There was no significant difference in mean scores (*p*-value > 0.05) for PHQ-9 (depression), GAD-7 (anxiety), WHO-5 (well-being), GSE (self-efficacy), BRIEF-COPE (adaptive and maladaptive coping behaviours) between both intervention and control groups (Table 2).

**Table 2:** Mean comparison between intervention and control group at baseline, T0

	Intervention (*N* = 96)		Control (*N* = 108)		Mean difference (95% CI)	*P*-value
	Mean	SD	Mean	SD		
PHQ-9	6.56	4.472	7.06	5.622	0.493 (-0.903, 1.889)	0.487
GAD-7	5.25	4.969	6.17	5.322	0.917 (-0.505, 2.338)	0.205
WHO-5	59.96	20.857	55.78	22.705	-4.181 (-10.195, 1.834)	0.172
GSE	28.74	5.478	29.16	5.332	0.418 (-1.079, 1.914)	0.582
Adaptive	1.85	0.570	1.85	0.516	-0.004 (-0.154, 0.147)	0.963
Maladaptive	0.91	0.551	0.89	0.537	-0.014 (-0.164, 0.137)	0.860

### Time-group interaction between intervention and control group

Table 3 shows the mean comparison and time-group interaction analysis for all assessments. Depression and anxiety score was significantly decreased across time among the intervention group (mean score = 6.56 at T0 vs. 5.77 at T3 and mean score = 5.25 at T0 vs. 4.80 at T3, respectively) and increased among the control group (mean score = 7.06 at T0 vs. 8.32 at T3 and mean score = 6.17 at T0 vs. 7.45 at T3 respectively). While self-efficacy and adaptive coping behaviour scores were increased by time among the intervention group (mean score = 28.74 at T0 vs. 29.76 at T3 and mean score = 1.85 at T0 vs. 1.89 at T3, respectively) and decreased among the control group (mean score = 29.16 at T0 vs. 27.88 at T3 and mean score = 1.85 at T0 vs. 1.68 at T3 respectively). Well-being and maladaptive coping strategies did not show any significant differences.

**Table 3:** Mental wellbeing changes and time-group interaction analysis

Tools		T0 - preassessment		T1 - Week 3		T2 - Week 5		T3 - Week 9		Within-group effect *P*-value	Between-group effect *P*-value	Time*Group Interaction effect *P*-value
		Mean	SD	Mean	SD	Mean	SD	Mean	SD			
PHQ-9												
	Intervention	6.56	4.472	7.05	5.284	5.74	4.397	5.77	4.827	0.097	0.014*	0.012*^a^
	Control	7.06	5.622	8.07	5.448	7.84	6.04	8.32	5.911			
GAD-7												
	Intervention	5.25	4.969	5.53	4.523	5.20	4.334	4.80	4.053	0.452	0.006*	0.025*^a^
	Control	6.17	5.322	6.72	5.406	7.08	5.848	7.45	5.173			
WHO-5												
	Intervention	59.96	20.857	61.90	20.291	66.16	17.802	66.25	18.740	0.017*	0.002*	0.051^a^
	Control	55.78	22.705	55.08	23.505	57.91	24.25	54.21	21.55			
GSE												
	Intervention	28.74	5.478	29.17	5.069	30.43	4.235	29.76	5.665	0.109	0.177	0.018*^a^
	Control	29.16	5.332	29.00	5.076	29.03	6.092	27.88	5.093			
Adaptive												
	Intervention	1.85	0.570	1.86	0.519	1.97	0.504	1.89	0.575	0.118	0.010*	0.018*^b^
	Control	1.85	0.516	1.74	0.481	1.72	0.580	1.68	0.547			
Maladaptive												
	Intervention	0.91	0.551	0.91	0.465	0.90	0.426	0.88	0.494	0.869	0.479	0.305^a^
	Control	0.89	0.537	0.93	0.503	0.95	0.584	0.99	0.563			

Intervention, *n* = 96; Control, *n* = 108

a. *P*-value is based on Greenhouse-Geisser

b. *P*-value is based on Sphericity Assumed

* significant time-group interaction/group effect

## Discussion

Our study found that junior doctors attending the *Sanubari* OHP program experienced significant reductions in depression and anxiety symptoms compared to the control group, as evidenced by decreased PHQ-9 and GAD-7 scores one-month post-intervention. The theoretical foundation of the *Sanubari* OHP encompasses various psychological and social principles, focusing on personal growth, resilience, and professional development [10]. Research indicates that individuals engaged in personal growth activities are better equipped to manage stress and adversity [26]. The *Sanubari* OHP fosters resilience by coaching participants to manage stress more effectively and encouraging personal development through targeted goals within their health domain. This focus on goal-setting and learning promotes a sense of achievement, which is associated with reduced depression and anxiety symptoms.

The findings also highlight significant improvements in self-efficacy and adaptive coping strategies among junior doctors in the intervention group compared to control group. This aligns with previous studies in Malaysia that have demonstrated the effectiveness of *Sanubari* OHP in enhancing self-efficacy and coping strategies [17]. A study among nurses demonstrated that *Sanubari* OHP significantly boosted self-efficacy and promoted positive coping strategies during the Covid-19 pandemic [16]. Early-career doctors face not only the challenge of adapting to new environments but also the development of self-identity and professional confidence. Self-efficacy, defined as the belief in one’s ability to achieve important goals, plays a crucial role in navigating these transitions [27]. According to self-efficacy theory, individuals build self-efficacy through four key sources: performance accomplishments, vicarious learning, verbal persuasion, and self-assessment of emotional and physiological responses [28, 29]. The *Sanubari* OHP is structured to facilitate learning and development through these sources, integrating healthy coping strategies like goal setting and problem-solving. Participants are encouraged to practice these skills between sessions through workbooks, enhancing their competence and self-efficacy in stress management.

The demanding nature of training to become a certified doctors places individuals in high-stress environments where emotional challenges are common and can lead to significant psychological strain [30]. During this period, understanding and applying adaptive coping strategies is crucial. Within the framework of *Sanubari* OHP, junior doctors explore both adaptive and non-adaptive coping mechanisms, learning how to manage stress in ways that support long-term well-being and professional success. Through the implementation of SMARTER goals—Specific, Measurable, Achievable, Relevant, Time-bound, Enjoyable, and Rewarding, junior doctors can systematically track their personal growth and maintain motivation. This goal-setting framework equips junior doctors with the tools to integrate healthy coping strategies during particularly stressful periods [31]. The wellness-based approach of *Sanubari* OHP emphasises positive mindset and well-being, shifting the focus from illness to growth [32]. Besides, within the framework of *Sanubari* OHP, which encompasses self-management programs requiring participants to reflect on their experiences and plan for their future, stress management techniques and social support are emphasised. Developing a strong support network and effective stress management strategies is essential for junior doctors, not only to navigate the challenges of their early careers but to achieve long-term professional and personal growth.

The findings showed that mean scores for depression and anxiety in the intervention group decreased from baseline to post-intervention; however, they increased back after one-month post-intervention. This pattern was also found for self-efficacy and adaptive coping strategies scores, which slightly decreased after one month. This could be explained by the stressful nature of work as junior doctors in public hospitals and the need for program sustainability. Therefore, it was recommended that a booster for the intervention package be administered after one month, which would aid participants in reflecting on the knowledge and skills they have acquired or practices they have adopted [17].

### Limitations and recommendations for future research

The study implemented a quasi-experimental design for psychological and the first virtual group psychological intervention research for junior doctors in Malaysia. The implications of this work are substantial, particularly in demonstrating the accessibility, flexibility, and cost-effectiveness of virtual interventions. Given the shift towards digital solutions in healthcare, our findings suggest that virtual group interventions can effectively reach a broader audience, reduce logistical barriers, and lower costs associated with in-person sessions. Nonetheless, the study’s results could not be regarded as representative of all the hospitals in Malaysia. The study assessed the Sanubari program’s effectiveness without considering variations among different departments and hospitals. A more extensive study with a larger and more diverse sample of hospitals and departments would ensure that the findings are more representative of the entire healthcare system in Malaysia. Researchers should also consider expanding the range of study variables, including psychological, behavioural, and environmental factors that may influence the outcomes of virtual interventions. Potential controlled variables could include internet connectivity quality, participant technological proficiency, and the degree of facilitator-participant interaction. Understanding these variables will help refine intervention designs and improve their efficacy.

## Conclusions

The *Sanubari* OHP represented a holistic strategy to enhance junior doctors’ self-efficacy and adaptive coping skills following the intervention. Consequently, the intervention also offers a thorough recovery-oriented method for reducing depression and anxiety. This intervention program has proven to have great potential and is very promising for improving mental well-being among junior doctors. The implication of the study is also perhaps to contribute as an evidence-based intervention for mental health care for junior doctors as well as a workplace mental health promotion.

## Data Availability

No datasets were generated or analysed during the current study.

## Abbreviations

GAD − 7: General Anxiety Disorders
GSE: General Self-Efficacy Scale
MREC: Medical Research Ethics Committee
OHP: Optimal Health Program
PHQ-9: Patient Health Questionnaire
WHO-5: WHO-5 Well-being Index
